# Sex-specific gut microbial and metabolic responses to inhaled diesel exhaust particle exposure are modified by probiotic treatment in C57BL/6 mice

**DOI:** 10.21203/rs.3.rs-10713368/v1

**Published:** 2026-08-27

**Authors:** Victoria Youngblood, Tyler D. Armstrong, Kayla Nguyen-Alley, Amaya E. Green, Molly E. Kelly, Bailee Johnson, Analana Stanley, Paisley Gilbreth, Mickaela Cook, Tallon Coxe, Jessica L. Bradshaw, Rajeev K. Azad, Rebecca L. Cunningham, Amie K. Lund

**Affiliations:** University of North Texas; University of North Texas; University of North Texas; University of North Texas; University of North Texas; University of North Texas; University of North Texas; University of North Texas; University of North Texas; University of North Texas; University of North Texas Health Science Center; University of North Texas; University of North Texas Health Science Center; University of North Texas

**Keywords:** Air pollution, Particulate matter, Gut microbiome, Short-chain fatty acids, Metabolic hormones, LPS, Sex differences

## Abstract

**Background:**

Epidemiological and experimental studies indicate that susceptibility to traffic-generated particulate matter (PM)-induced metabolic dysfunction varies by sex, with females frequently exhibiting greater vulnerability. Recent evidence suggests that disruption of the lung-gut axis and subsequent gut-derived inflammatory signaling may contribute to these outcomes. However, the effects of inhaled PM on gut microbiome signaling and the metabolic milieu across sexes remain inadequately characterized. This study examined whether diesel exhaust particulate (DEP) exposure induces sex-specific metabolic and inflammatory responses and whether probiotic supplementation differentially modifies these effects in males and females.

**Methods:**

Male and female C57BL/6 mice were exposed to 35 μg DEP (1 mg/mL, SRM-2975) or saline control via oropharyngeal aspiration twice weekly for 50 days, with or without probiotic supplementation (Winclove Ecologic^®^ Barrier probiotics). Systemic metabolic outcomes in plasma were assessed using a multiplex hormone panel. Gut microbiome composition was characterized using 16S rRNA sequencing, and gut-derived signaling was evaluated through plasma lipopolysaccharide (LPS) quantification and short-chain fatty acid (SCFA) analysis. Three-way ANOVAs with sex as a biological variable were conducted to determine differential responses to DEP exposure and probiotic intervention.

**Results:**

DEP exposure induced sex-dependent alterations in gut microbial composition, circulating metabolites, and endotoxemia. In females, DEP exposure resulted in taxonomic shifts and reduced microbial diversity, whereas probiotic treatment produced the most pronounced community-level expansion in DEP-exposed males. Beta-diversity analyses confirmed significant treatment-associated differences in community composition, identifying probiotic treatment as the primary driver, with stronger community-level effects in females and more limited effects in males. These changes corresponded with alterations in circulating SCFA and LPS levels, with more pronounced effects observed in females.

**Conclusions:**

Biological sex is a major determinant of susceptibility to DEP-induced metabolic and inflammatory dysregulation. Females demonstrated heightened systemic and gut-derived responses to DEP exposure. Probiotic supplementation modified several of these functions, with effects varying by sex. Notably, probiotic supplementation produced opposing effects on glucagon in DEP-exposed animals depending on sex, underscoring the importance of biological sex in determining both the direction and magnitude of microbiome-targeted intervention outcomes. These findings support the inclusion of sex as a biological variable in environmental health research and suggest that microbiome-targeted interventions may provide sex-specific protective effects against PM-induced metabolic dysfunction.

## Plain English Summary

Exposure to air pollution from diesel engine exhaust has been linked to metabolic and inflammatory diseases, but these responses can differ across biological sexes. In this study, we investigated whether diesel exhaust particles (DEP) affect metabolism and the gut microbiome differently in males and females and whether probiotics can modify these effects. For this study, both male and female mice were exposed to DEP over 50 days, with or without probiotic supplementation. We then examined changes in blood hormone levels and other metabolic markers, gut bacteria profiles, and substances produced by gut microbes that can influence inflammation and overall health. We observed that DEP exposure caused different changes in the gut microbiome and metabolism of male and female mice. Females generally showed greater disruption, including reduced gut bacteria diversity and larger changes in inflammatory and metabolic factors produced by the gut bacteria. Probiotic treatment also altered the gut microbiome and metabolic responses, but the effects differed between males and females. For example, we observed opposite effects of DEP-exposure on the hormone glucagon, which maintains blood glucose levels, in males vs. females. These findings show that biological sex can strongly influence how the body responds to diesel-related air pollution. They also suggest that interventions targeting the gut microbiome, such as probiotics, may not affect males and females in the same way. Understanding these sex-specific responses may help improve strategies for preventing or reducing the metabolic and inflammatory health effects associated with air pollution.

## Introduction

The World Health Organization estimates that ambient air pollution exposure is associated with 4.2 million premature deaths annually (1). Numerous epidemiological studies have linked traffic-generated particulate matter (PM) to adverse health outcomes, including cardiovascular, neurological, and metabolic diseases (2–4). Although the adverse respiratory effects of air pollution are well established, growing evidence indicates that inhaled PM exerts systemic effects beyond the lungs, influencing physiological outcomes through inflammatory and metabolic pathways (5, 6). Emerging evidence also suggests that susceptibility to these adverse outcomes differs between males and females, highlighting the importance of understanding sex-specific biological responses to environmental exposures (7–9).

One mechanism by which inhaled PM may influence systemic health is through the lung-gut axis. Inhaled particles can reach the gastrointestinal tract via mucociliary clearance and subsequent swallowing, providing a direct route for pollutants to alter intestinal physiology and microbial communities (10). While previous studies have primarily examined the effects of ingested PM on the gut microbiome, fewer have investigated how inhaled PM disrupts gut homeostasis through the lung-gut axis (11, 12). Experimental studies demonstrate that inhaled PM compromises gut barrier integrity, alters microbial composition, and promotes endotoxin translocation, collectively contributing to systemic inflammatory signaling (13–15). These findings suggest that PM-induced alterations in the gut microbiome may influence host metabolism by modifying microbial-derived signaling molecules.

The gut microbiome plays a central role in maintaining host metabolic homeostasis through the production of microbial-derived metabolites that regulate host immunity, energy balance, and glucose metabolism (16). Previous work from our laboratory has demonstrated that PM exposure alters gut microbial composition, including reductions in beneficial taxa such as Turicibacter and members of the Lachnospiraceae family, which are important producers of short-chain fatty acids (SCFAs) (14, 17). Concurrently, PM exposure increases gram-negative taxa, including *Desulfovibrio*, which are associated with lipopolysaccharide (LPS) endotoxin production and translocation resulting in systemic inflammation (18).

Among gut-derived metabolites, SCFAs such as acetate and butyrate exert anti-inflammatory effects by strengthening intestinal barrier integrity, supporting immune regulation, and limiting systemic endotoxin exposure (19). In contrast, LPS is a potent pro-inflammatory endotoxin that promotes metabolic endotoxemia and activates inflammatory signaling through toll-like receptor 4 (TLR4) (20). Modulation of these microbial-derived signaling pathways has been implicated in the development of systemic metabolic dysfunction and may represent a mechanistic link between environmental exposures and adverse metabolic outcomes (21).

Probiotics, defined as living microorganisms that confer a health benefit to the host, can reshape gut microbial composition and regulate the production of microbial-derived metabolites, making them a promising strategy for mitigating environmentally induced metabolic dysfunction (22, 23). Previous findings from our laboratory demonstrated that probiotic supplementation restores beneficial SCFA-producing gut microbial populations and reduces circulating LPS following PM exposure (14). Through these mechanisms, probiotic supplementation may enhance SCFA production, strengthen gut barrier integrity, and reduce circulating endotoxin load and systemic inflammatory burden. However, whether probiotic-mediated protection differs between males and females remains poorly understood.

Approximately 88% of adults in the United States fail to meet the criteria for optimal metabolic health (24). Characteristics of metabolic dysfunction include impaired glucose homeostasis, dysregulated lipid metabolism, altered energy balance, and chronic low-grade inflammation. These abnormalities collectively contribute to the development of cardiometabolic diseases such as obesity, insulin resistance, and type 2 diabetes. Additionally, many of these disturbances arise from crosstalk between immune and endocrine signaling pathways, which play a critical role in regulating systemic metabolic function. Accumulating evidence from both epidemiological and animal studies links traffic-related air pollution and PM exposure to systemic inflammation and adverse metabolic outcomes (4). Human and animal studies have reported sex-specific responses to PM exposure, with females often exhibiting greater susceptibility to environmentally induced inflammatory and metabolic disturbances (7, 8, 25). These differences are thought to be driven by sex-specific variations in hormonal signaling, immune function, and metabolic regulation. However, the biological mechanisms and sex-specific differences by which PM exposure drives systemic metabolic dysfunction, particularly those involving gut-derived signaling pathways, remain uncharacterized.

Despite growing evidence that inhaled PM disrupts the gut microbiome and contributes to metabolic dysfunction, the extent to which these lung–gut interactions differ by biological sex remains largely unknown. Likewise, sex-specific differences in probiotic-mediated protection have not been systematically investigated. We therefore hypothesized that biological sex is a key determinant of gut microbiome remodeling, microbial-derived signaling, and systemic metabolic responses following diesel exhaust particle (DEP) exposure, and that probiotic supplementation would differentially modulate these responses in males and females. The objective of this study was to characterize sex-specific differences in gut microbiome composition, microbial signaling, and systemic metabolic outcomes following DEP exposure in mice.

## Materials and Methods

### Animal care and exposure.

Four-to-six-week-old wild-type C57BL/6NTac (Taconic) female and male mice were administered a combination of norfloxacin (1g/L; Sigma, N98900) and ampicillin (1 g/L; Sigma, A0166) in drinking water for 2 weeks to reduce baseline gut microbial variability. Following antibiotic treatment, mice were randomly assigned to exposure (DEP or saline) and treatment (probiotic or placebo) groups (Table 1). Mice assigned to DEP exposure groups received 35 μg of DEP (NIST Standard reference material #2975) suspended in 35 μl of 0.9% sterile saline via oropharyngeal aspiration (OA). Control mice received OA of sterile saline only. Exposures were administered twice weekly for 50 days. Immediately prior to each exposure, DEP mixtures were suspended in saline and sonicated for 5 minutes to ensure particle dispersion. For OA administration, mice were anesthetized with 2% isoflurane (Butler Schein Animal Health) and positioned at approximately a 60° angle using a rodent intubation stand (Biolite RIS 100). Body weights were taken weekly throughout the study and the delta from day 0 to day 50 were used for body weight change analyses. All experimental procedures were approved by the University of North Texas Institutional Animal Care and Use Committee (IACUC) under protocol 22014 and follow the Guide for the Care and Use of Laboratory Animals (NIH Publication No. 85 – 23, rev. 1996).

## Probiotic treatment

Mice from each exposure group were assigned to receive either 0.3 g/day of Ecologic® Barrier 849 probiotic formulation (approximately 7.5 × 10^8^ CFU/day; Winclove Probiotics B.V., Amsterdam, The Netherlands), or nutrient matched placebo (Winclove Probiotics), administered in drinking water throughout the DEP exposure study (PRO, *n* = *12*). Ecologic® Barrier 849 is a proprietary probiotic blend consisting of nine bacterial strains: *Bifidobacterium bifidum* W23, *B. lactis* W51, *B. lactis* W52, *Lactobacillus acidophilus* W37, *L. brevis* W63, *L. casei* W56, *L. salvarius* W24, *Lactococci lactis* W19, and *Lc. lacis* W58 in a carrier matrix of maize starch, maltodextrin, and minerals. Probiotics (and placebo) were provided in low-drip metered water bottles to minimize spillage and allow monitoring to adjust dosing to maintain consistent probiotic or placebo consumption across experimental groups over the course of the study. Experimental group definitions and abbreviations used throughout the manuscript are summarized in Table 1.

## Tissue and blood collection

Mice were fasted overnight prior to tissue collection; however, probiotic or placebo remained in the drinking water. Mice were anesthetized and euthanized by exsanguination via cardiac puncture within 24 h of final DEP exposure. Whole blood was collected in heparinized tubes and centrifuged at 4°C at 3,800 x g for 10 minutes. The resulting supernatant plasma was collected and stored at −80°C. Plasma samples were later treated with a 1:1 ratio of HemogloBind (Biotech Support Group LLC, East Brunswick, NJ #HO145) to remove hemoglobin without disrupting proteins and analytes and stored again at −80°C. Blood glucose levels were measured at the time of sacrifice using tail vein sampling. A small incision was made at the tip of the tail using a sterile lancet, and a drop of blood was collected onto a test strip for immediate measurement with a handheld glucometer (CareSens N Blood Glucose Monitor, I-Sens Inc., Seoul, Korea). For stool collections, two-centimeter segments from the mid-duodenum, jejunum, and ileum were excised, and luminal contents were retained and stored at −80°C.

## Circulating LPS

Circulating LPS levels were measured in plasma using Pierce Chromogenic endotoxin Quant Kit (ThermoFisher Scientific, Waltham, MA), following the manufacturer’s protocol. Plasma samples were diluted 1:100 dilution in endotoxin-free water and run in duplicate. Absorbance was measured using a multimode plate reader (Take-3 plate, Cytation 5, Bio-Tek, El Segundo, CA). LPS concentrations were calculated from the standard curve using a four-parameter logistic (4PL) regression model and are reported as endotoxin units per milliliter (EU/mL).

## Tissue homogenization and DNA extraction

Microbial DNA was homogenized and extracted using the Quick-DNA Fecal/Soil Microbe MiniPrep Kit (Zymo Research, Irvine, CA, USA) according to the manufacturer’s protocol. Samples were quantified and purity confirmed via UV-VIS spectrometry (Cytation 5, Bio-Tek, El Segundo, CA) at 260 nm absorbance. Samples were diluted to 20 ng/sample in DNA Elution Buffer in preparation for 16S rRNA sequencing.

### 16S rRNA gene sequencing

DNA samples were processed via 16S next-generation sequencing in the UNT Genomics Center (Denton, TX). DNA was converted into 16S amplicon libraries using the Zymo Quick-16S Plus NGS Library Prep Kit. Library concentrations and fragment sizes were assessed on an Agilent 4200 TapeStation, normalized, and sequenced on an Illumina MiSeq using the MiSeq Reagent Kit v3 (600-cycle; 2×300 bp).

## Bioinformatic analysis and taxonomic classification

Paired-end FASTQ files were imported into QIIME 2 (v2024.10) using a sample manifest (26). Cutadapt (v4.9) was used to remove V3–V4 primer sequences (forward: CCTACGGGNGGCWGCAG; reverse: GACTACNVGGGTATCTAATCC) using default parameters (10% error rate) (27). DADA2 (via q2-dada2 v2024.10.0) was used for quality filtering, denoising, paired-end read merging, and chimera removal, with forward and reverse reads truncated to 240 bp and 200 bp, respectively (28). Parameters for max expected errors and chimera removal remained at their standard default settings. Amplicon sequence variants (ASVs) were mapped to the Greengenes2 2024.09 reference backbone using the q2-greengenes2 non-v4-16s parameter, and taxonomy was assigned using the Greengenes2 phylogenetic taxonomy-from-table classifier (29). The Greengenes2 2024.09 reference phylogeny was used for phylogenetic diversity analyses.

Samples were rarefied to a depth of 6,389 reads prior to downstream diversity analyses. A depth of 6,389 reads represented the smallest library size in the dataset, ensuring no exclusion of samples in our analysis; rarefaction curves allowed for the optimization of this depth for capturing maximum sample richness. Alpha diversity was calculated as Chao1, ACE, Shannon entropy, and Simpson evenness from the rarefied feature table. Beta diversity was calculated using weighted and unweighted UniFrac, Bray–Curtis, and Jaccard distance metrics. Principal coordinates analysis (PCoA) was used to visualize beta diversity ordinations. Relative abundance was summarized at the phylum and genus levels after collapsing the Greengenes2 feature table.

For 16S rRNA gene sequencing data, alpha diversity metrics (Chao1, ACE, Shannon entropy, and Simpson evenness) were compared across the four experimental groups (CON-PLC, CON-PRO, DEP-PLC, and DEP-PRO) using one-way ANOVA when the normality assumption was met for the data (Shapiro–Wilk test) or Kruskal–Wallis test when the assumption was not met. Pairwise comparisons of alpha diversity between groups were performed using two-sided Mann–Whitney U tests. Beta diversity was assessed using PERMANOVA and ANOSIM on weighted UniFrac, unweighted UniFrac, Bray–Curtis, and Jaccard distance matrices with 9,999 permutations. Pairwise PERMANOVA with Benjamini–Hochberg false discovery rate (FDR) correction was performed for selected group comparisons. Differential taxonomic abundance at the phylum and genus levels was evaluated using two-sided Mann–Whitney U test with Benjamini–Hochberg false discovery rate (FDR) correction. Microbiome statistical analyses were performed in Python 3.10.14 using SciPy (1.10.1), scikit-bio (0.6.0), and statsmodels (0.14.4) (30).

## Short-chain fatty acid quantification

Derivatized SCFAs from plasma were separated using an Agilent 1290 Infinity II liquid chromatography system. The extracts were kept at 5°C in an autosampler. The metabolites were resolved at 45°C using a reverse phase C18 Kinetex column (2.1 x 100 mm; 2.6 mm) associated with one inline filter frit (0.3 mm) from Agilent. The liquid chromatography gradient was made of 0.1% (v/v) formic acid + 10 mM ammonium formate in water (A) and 0.1% (v/v) formic acid in methanol/isopropanol (90:10; v/v) (B). The total LC-MS/MS run was 6.5 minutes with a flow rate of 400 mL/min. The following gradient was applied to separate the compounds: B = 0 minute 32%, 4 minutes 60%, 4-4.8 minutes 65%, 4.8–4.9 minutes 98%, 4.9–5.2 min 98%, 5.2–5.3 minutes 32%, 5.3–6.5 minutes 32%. The injection needle was rinsed with 50% aqueous methanol. 5 mL of extracts or 0.5 mL external standard mixture was injected onto the column.

Detection was performed on an AB Sciex hybrid Triple Quadrupole/Ion trap mass spectrometer (QTRAP) 6500+. Electrospray positive ionization was used to acquire mass spectra of metabolites. Analytes were simultaneously detected as precursor ion/product ion pair using multiple reaction monitoring (MRM). The source parameters such as curtain gas, temperature, nebulizer gas (GS1), heating gas (GS2), and collision-activated dissociation (CAD) were kept constant during MRM survey scan. The dwell time in the mass spectrometer was set to 25 msec. Analyst 1.7 and MultiQuant vs 3.0 software from AB Sciex were utilized to acquire and process the LC-MS/MS data, respectively. Derivatized SCFAs were identified and quantified using a mixture of known external standards analyzed alongside biological samples.

## Metabolic biomarkers

Levels of metabolic hormones and adipokines were quantified in plasma using a MILLIPLEX Mouse Metabolic Hormone Expanded Panel (Sigma Millipore, MMHE-44K) following manufacturer protocols for plasma samples. Standards, quality controls, and diluted plasma samples were plated in duplicate and incubated for 18 hours overnight with coupled magnetic beads. Samples were measured on a Luminex® 200^™^ instrument and quantification was performed using a five-parameter logistic curve analysis in xPONENT® software version 4.3 (Luminex Corporation, Austin, TX, USA). Quality control values for all analytes were within the range provided by the manufacturer.

## Statistical Analysis

For all other data analysis not previously described, a three-way ANOVA was used to assess the effects of sex, exposure, and probiotic treatment, as well as their interactions. Data are expressed as mean ± SEM followed by a post-hoc Holm–Šídák’s multiple comparisons test. Statistical analysis was conducted, and graphs were generated using GraphPad Prism (Version 10.6.0). A p-value < 0.050 was considered statistically significant.

## Results

### DEP exposure and probiotic supplementation alter microbial diversity in a sex-dependent manner.

**Figure 1:**
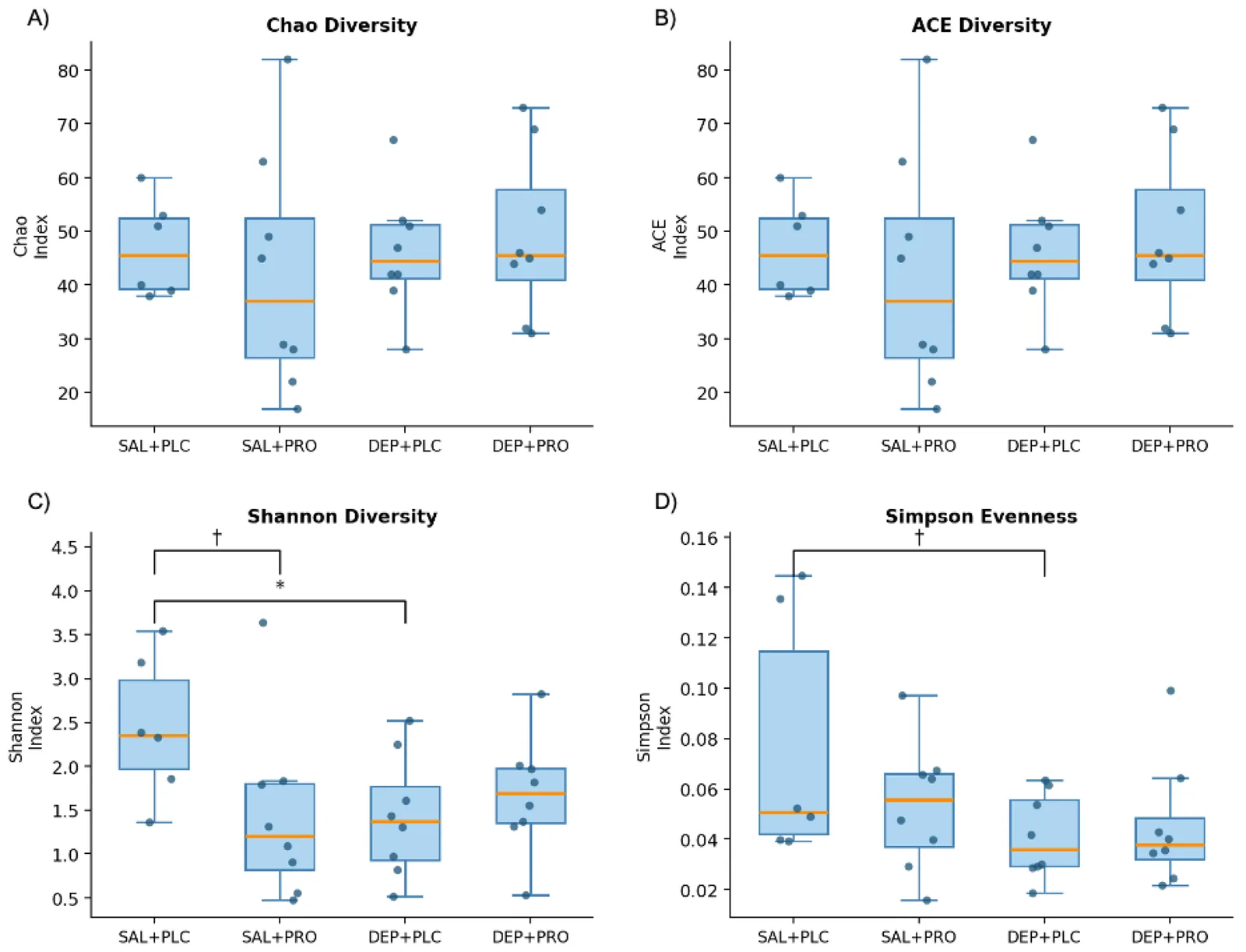
Alpha diversity of gut microbial communities across exposure and treatment in females. Gut microbiome alpha diversity measurements in female C57BL/6 mice following diesel exhaust particle (DEP) exposure and probiotic treatment, including richness metrics (Chao and ACE) and diversity metrics (Shannon and Simpson evenness). Mice were exposed to 35 μg DEP suspended in saline or sterile saline control, administered 2×/week for 50 days, and treated with placebo (PLC) or probiotic (PRO). Data are presented as box-and-whisker plots showing median and interquartile range with individual values overlaid. Statistical significance was determined by two-way ANOVA followed by appropriate multiple-comparisons testing. Brackets indicate significant or trending differences: †p < 0.10, *p < 0.05.

**Figure 2:**
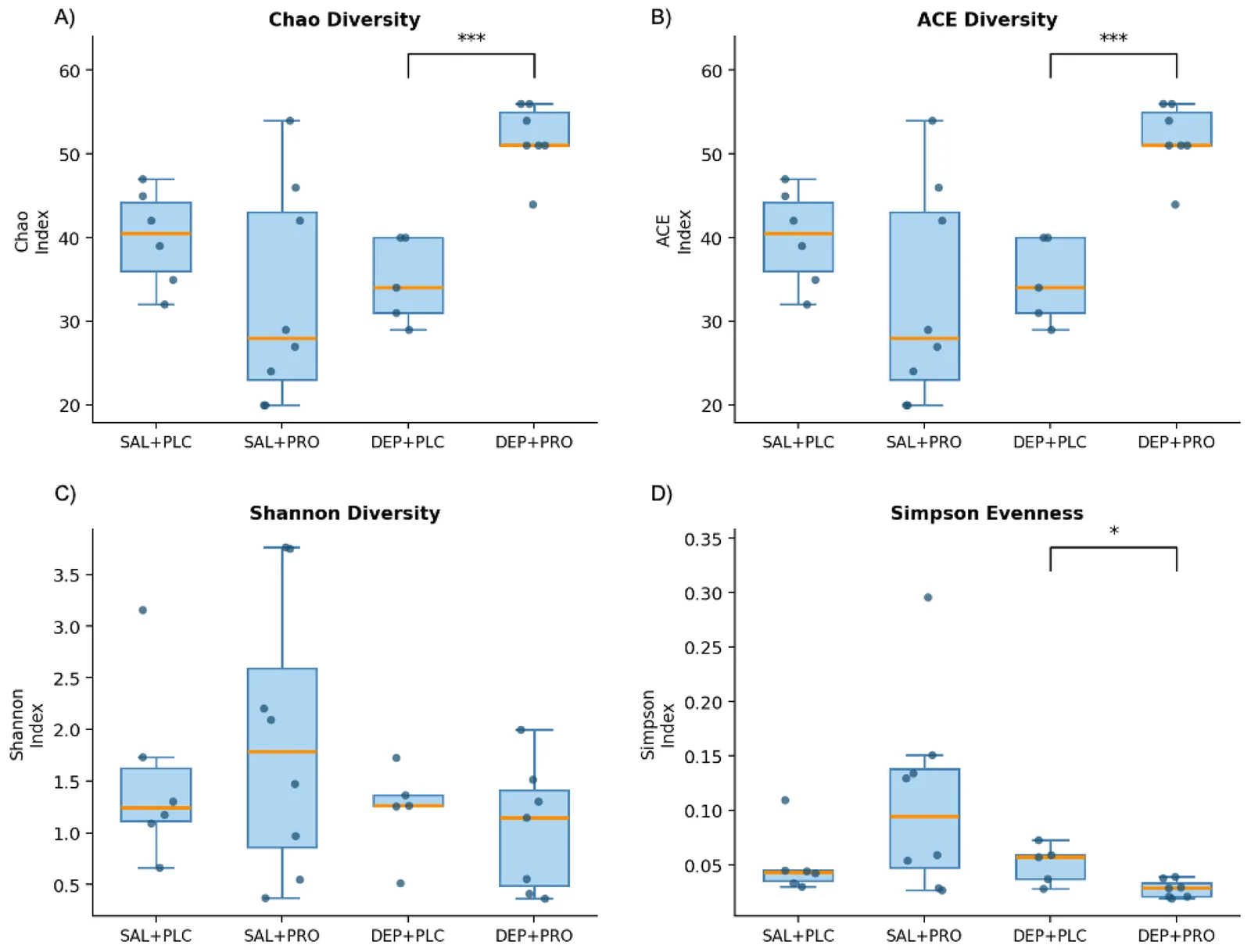
Alpha diversity of gut microbial communities across exposure and treatment in males. Gut microbiome alpha diversity measurements in in male C57BL/6 mice following diesel exhaust particle (DEP) exposure and probiotic treatment, including richness metrics (Chao and ACE) and diversity metrics (Shannon and Simpson evenness). Mice were exposed to 35 μg DEP suspended in saline or sterile saline control, administered 2×/week for 50 days, and treated with placebo (PLC) or probiotic (PRO). Data are presented as box-and-whisker plots showing median and interquartile range with individual values overlaid. Statistical significance was determined by two-way ANOVA followed by appropriate multiple-comparison testing. Brackets indicate statistically significant or trending differences: *p < 0.05, ***p < 0.001.

We calculated the α-diversity of microbial profiles in male and female mice, as determined by richness (Chao and ACE) and diversity (Shannon and Simpson evenness). In females (Fig. 1) Shannon diversity was significantly reduced in DEP + PLC animals compared to SAL + PLC controls (p = 0.026), and a similar trend was observed for Simpson evenness (p = 0.078), indicating that DEP exposure was associated with reduced microbial diversity and community evenness in females. In males (Fig. 2), overall group comparisons revealed significant differences in richness (Chao1: F = 7.36, p = 0.0014; ACE: F = 7.36, p = 0.0014) and Simpson evenness (F = 3.27, p = 0.041). Pairwise analysis revealed that probiotic supplementation significantly increased species richness in DEP-exposed animals, with DEP + PRO animals showing markedly higher Chao1 and ACE estimates compared to the DEP + PLC group (p = 0.0001). Simpson evenness was also significantly higher in DEP + PRO compared to DEP + PLC males (p = 0.013), suggesting that probiotic treatment restored community evenness specifically in the context of DEP exposure. Together, these findings indicated sex-specific patterns of α-diversity response, with DEP reducing diversity in females while probiotic supplementation expanded richness and evenness in DEP-exposed males.

**Figure 3:**
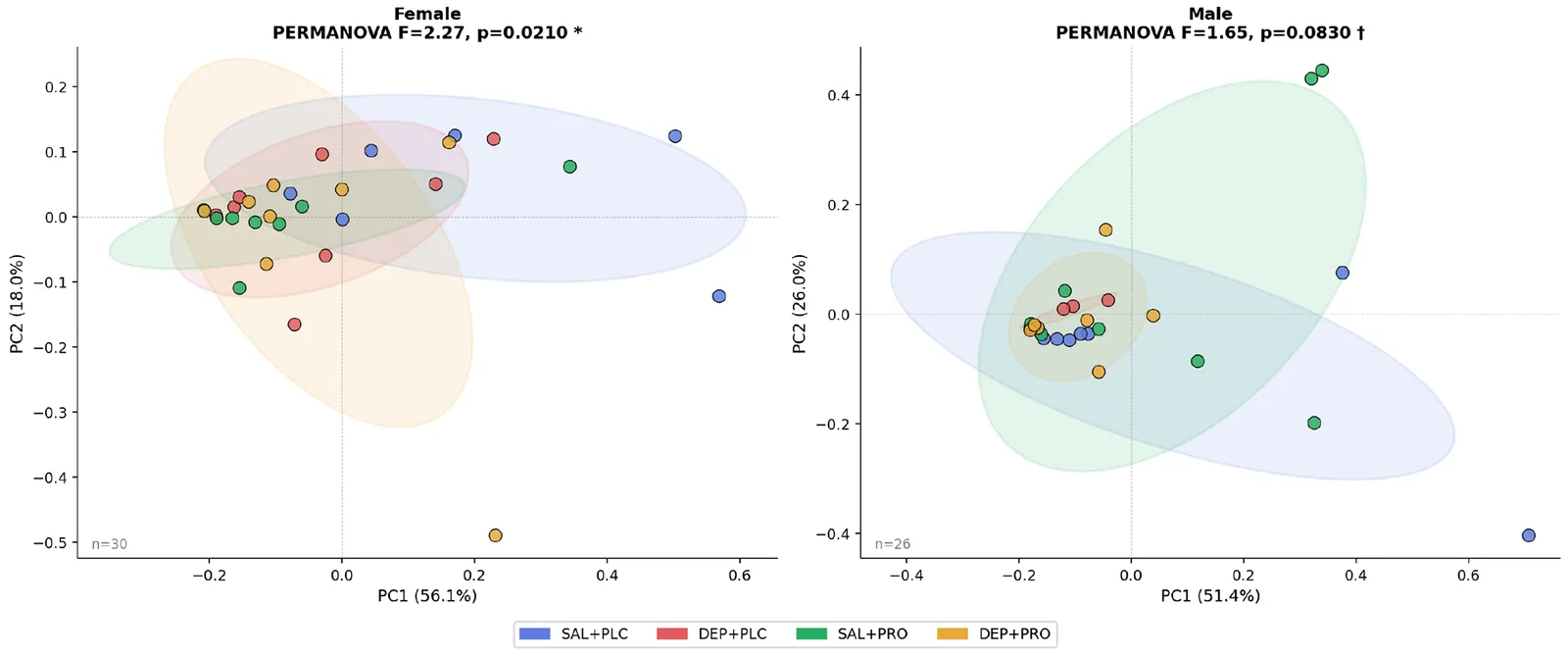
Beta diversity of gut microbial communities across exposure, treatment and sex. Beta diversity in the gut microbiome profiles in female and male C57BL/6 mice following diesel exhaust particle (DEP) exposure and probiotic treatment. Principal coordinates analysis (PCoA) plot Bray–Curtis distances for females (right) and males (left). Mice were exposed to 35 μg DEP suspended in saline or sterile saline control, administered 2×/week for 50 days, and treated with placebo (PLC) or probiotic (PRO). Each point represents an individual animal. Axes indicate the percentage of variance explained by each principal coordinate. Statistical significance was assessed by PERMANOVA.

We next evaluated β-diversity using weighted UniFrac, unweighted UniFrac, and Bray–Curtis PCoA plots (Fig. 3). Beta diversity analysis revealed that probiotic treatment, but not DEP exposure, significantly altered gut microbiome composition. In females, PERMANOVA analysis using multiple distance metrics demonstrated significant differences across 4 treatment groups (unweighted UniFrac: F = 2.07, p = 0.0006; Bray–Curtis: F = 2.05, p = 0.0014; Jaccard: F = 1.94, p = 0.0004). Probiotic treatment was consistently associated with significant alterations in microbial composition across all distance metrics (unweighted UniFrac: F = 3.85, p = 0.0004; Bray–Curtis: F = 2.24, p = 0.0226; Jaccard: F = 2.93, p = 0.0004). In contrast, DEP exposure alone did not significantly alter community structure. This suggests that exposure alone had a limited impact on overall microbiome community structure. Additionally, ANOSIM analysis, consistent with the PERMANOVA results, revealed significant differences associated with probiotic treatment and overall group comparisons in females, with no significant DEP effects.

## Sex-specific alterations in gut microbial composition following DEP exposure and probiotic treatment

**Figure 4:**
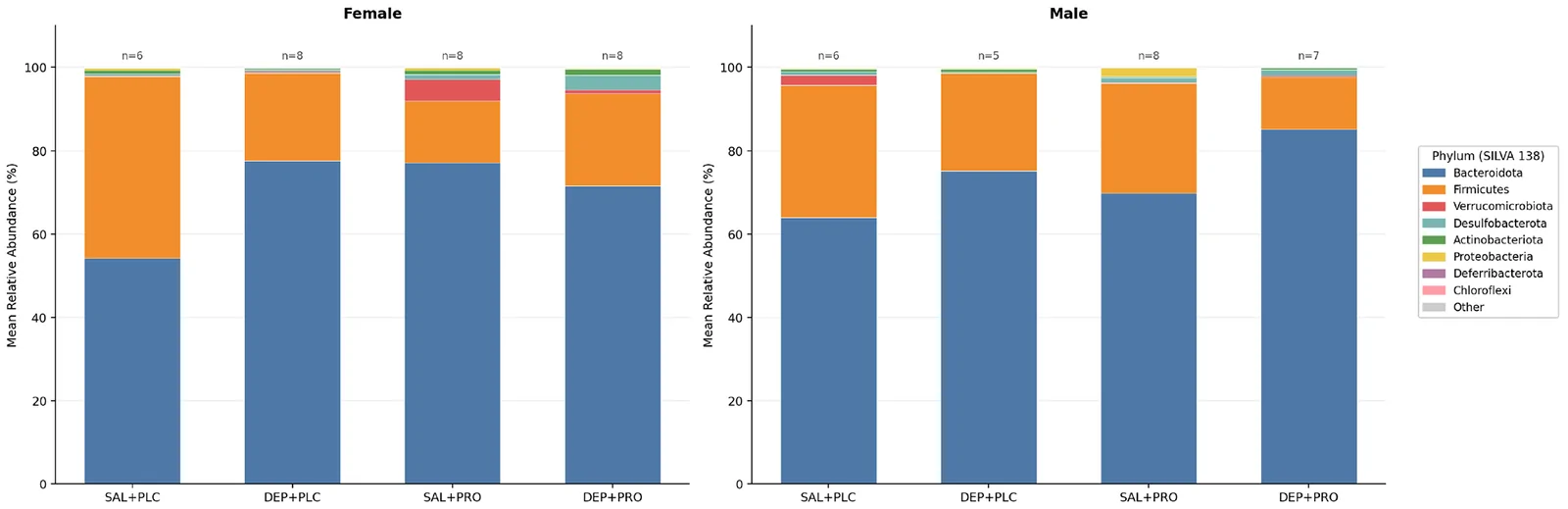
Phylum level relative taxa abundance across exposure, treatment, and sex. Data shows normalized 100% relative abundance for top 8 most abundant phyla stacked bar graph with percentages within the small intestine in C57BL/6 female **(A)** or male**(B)** mice. Mice were exposed to 35 μg DEP suspended in saline or sterile saline control, administered 2×/week for 50 days, and treated with placebo (PLC) or probiotic (PRO).

To characterize the sex-specific effects of inhaled DEP and probiotic supplementation on the gut microbial profiles, we determined the relative abundance of bacterial in the small intestines of both male and female C57BL/6 mice. Figure 4 shows the relative abundance and relative percentage out of 100% of the top phyla in both male and female mice exposed to inhaled DEP or saline controls (SAL), supplemented with probiotics (PRO) or a nutrient-matched placebo (PLC). At the phylum level, we observed that the gut microbiome across all treatment groups was dominated by Bacteroidota and Firmicutes. In males, Bacteroidota accounted for approximately 64.0% of the microbiome in the SAL + PLC animals and increased with DEP exposure to 75.1%. Probiotic treatment had more pronounced effects than DEP in males, increasing Bacteroidota to 85.1% and decreasing Firmicutes to 12.7% in DEP + PRO animals. In contrast, females exhibited a more balanced baseline microbiome, with Bacteroidota at 54.2% and Firmicutes at 43.7% in SAL + PLC mice. Following DEP exposure, females show a pronounced shift towards Bacteroidota dominance, increasing by 23.4%, accompanied by a reduction in Firmicutes by 22.6% in DEP + PLC mice. Probiotic supplementation further altered phylum composition in females, increasing Bacteroidota by 22.9% in SAL + PRO animals, while reducing Firmicutes by 28.9%.

**Figure 5:**
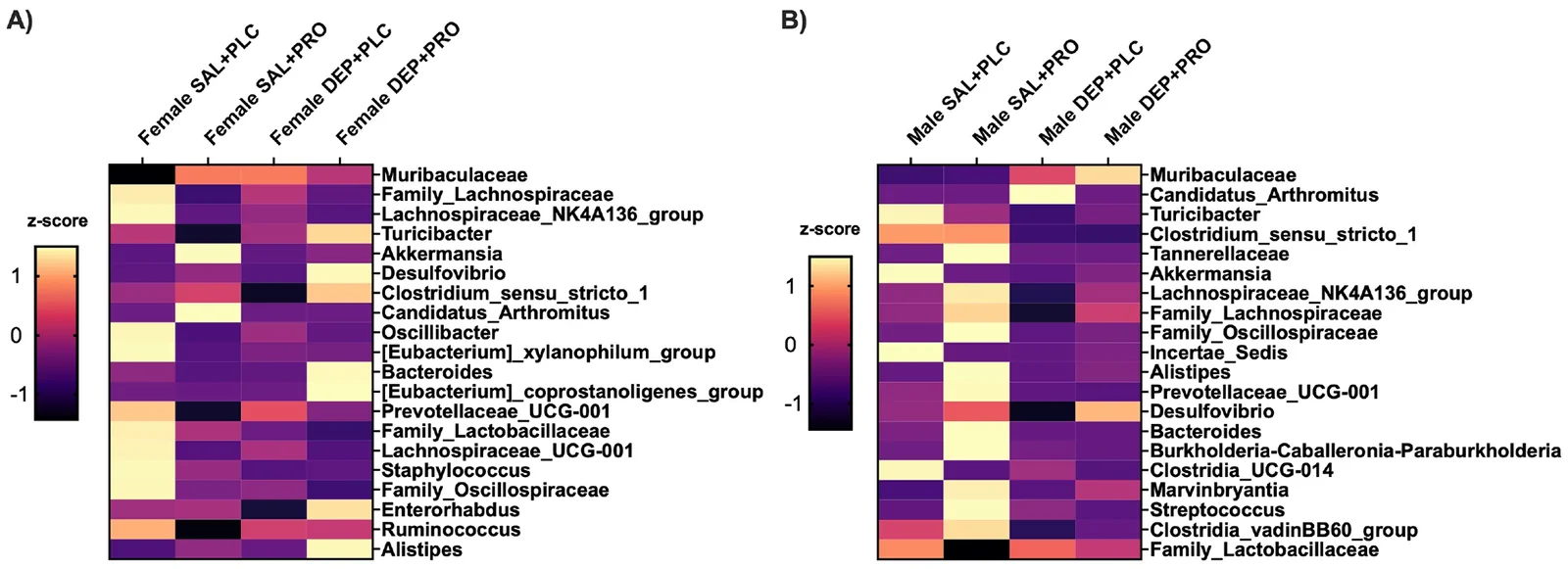
Heatmap of relative abundance across exposure, treatment, and sex. Heatmaps of the relative abundance of selected gut bacterial taxa in female and male C57BL/6 mice following diesel exhaust particle (DEP) exposure and probiotic treatment. Heatmaps show z-score–normalized relative abundance of taxa across treatment groups in (A) females (B) and males. Mice were exposed to 35 μg DEP suspended in saline or sterile saline control, administered 2×/week for 50 days, and treated with placebo (PLC) or probiotic (PRO). Columns represent treatment groups (SAL+PLC, SAL+PRO, DEP+PLC, and DEP+PRO), and rows represent bacterial taxa. Warmer colors (oranges, yellow) indicate higher relative abundance, whereas cooler colors (purples, black) indicate lower relative abundance relative to the group mean.

**Figure 6:**
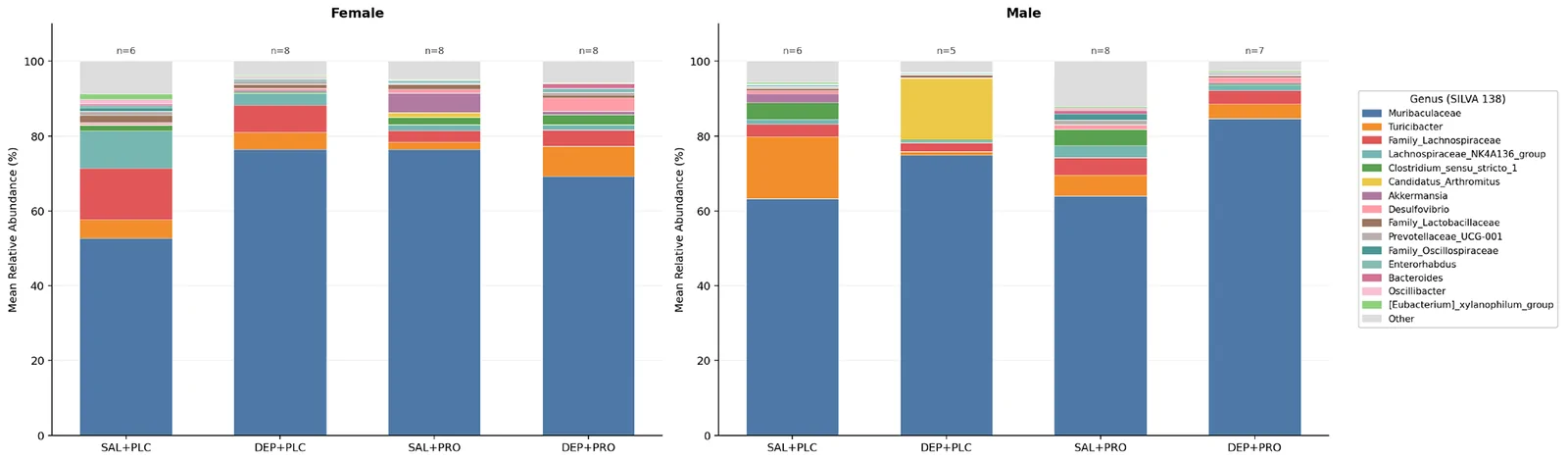
Genus level relative taxa abundance across exposure, treatment, and sex. Data shows normalized 100% relative abundance for top 15 most abundant genus stacked bar graph with percentages within the small intestine in C57BL/6 female **(A)** or male **(B)** mice. Mice were exposed to 35 μg DEP suspended in saline or sterile saline control, administered 2×/week for 50 days, and treated with placebo (PLC) or probiotic (PRO).

Genus-level composition reflected these phylum-level shifts (Fig. 5). In female mice (Fig. 6A), several taxa displayed treatment-associated shifts. *Muribaculaceae* abundance was reduced in SAL + PRO animals but increased following DEP exposure. In contrast, *Turinacter* and *Clostridium sensu stricto 1* showed reduced relative abundance following probiotic supplementation and DEP exposure. Family_*Lachnospiraceae* dropped substantially from SAL + PLC (13.7%) to DEP + PLC (7.2%). Several low-relative abundance genera, including *Desulfovibrio* and *Enterorhabdus* exhibited treatment-specific variation across groups.

In male mice (Fig. 6B), microbial responses differed from those observed in females. DEP exposure was associated with reduced relative abundance of *Turicibacter* and *Clostridium sensu stricto 1*, while *Muribaculaceae* abundance increased in DEP + PRO animals. The reduction in *Turicibacter* is substantial, dropping 15.6% with DEP exposure. Additional genera including *Prevotellaceae UCG-001*, *Marvinbryantia*, and *Alistipes* displayed increased relative abundance following probiotic treatment. Notably, *Candidatus_Arthromitus* was enriched specifically in DEP + PLC males. Together, these results demonstrate sex-specific alterations in genus-level microbial composition following DEP exposure, with probiotic treatment modifying several of these microbial shifts.

### Short-chain and branched-chain fatty acid profiles are differentially modulated by exposure and sex.

**Figure 7:**
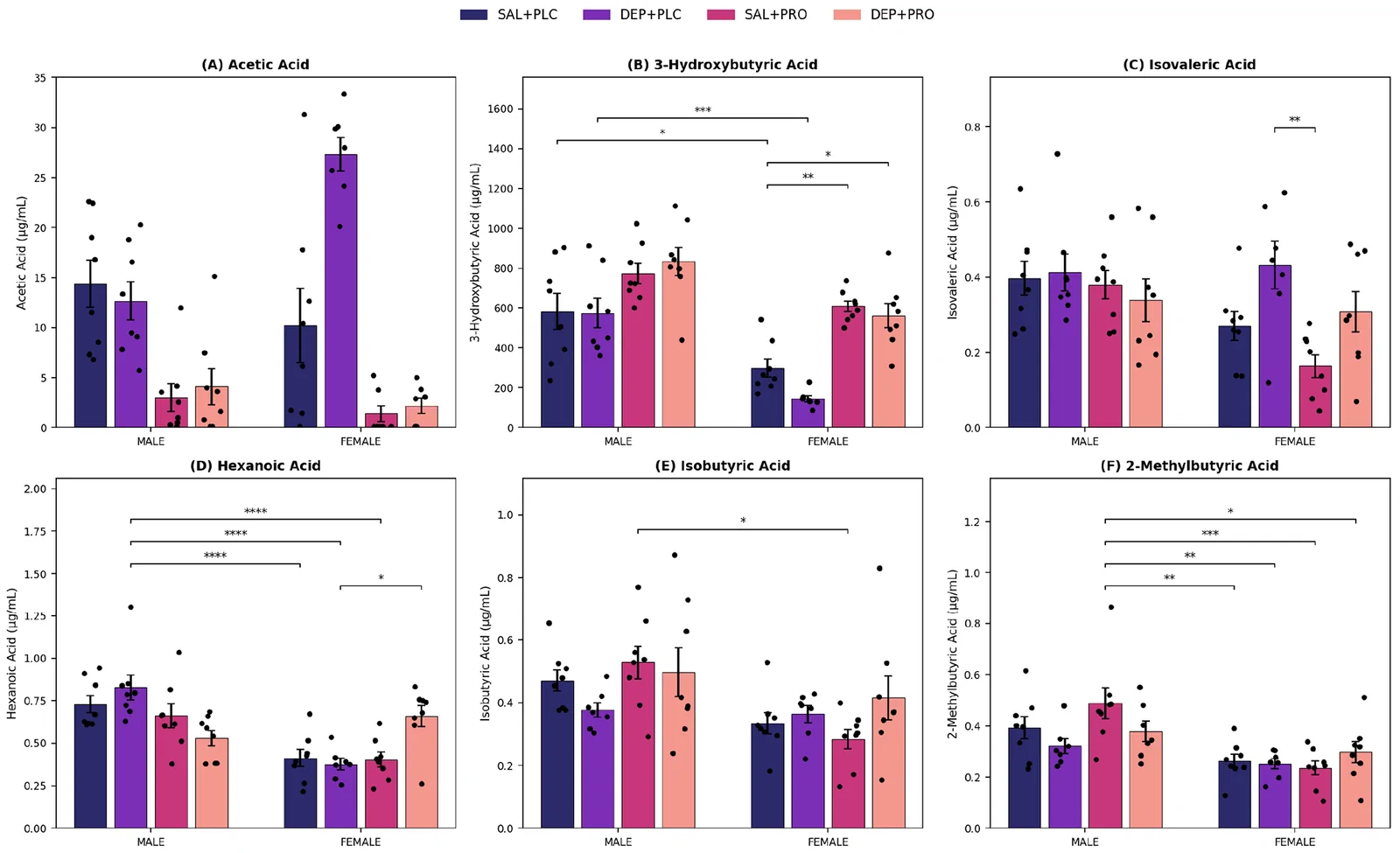
Plasma short-chain fatty acid (SCFA) concentrations across exposure, treatment, and sex. Circulating plasma concentrations of (A) acetic acid, (B) 3-hydroxybutyric acid, (C) isovaleric acid, (D) hexanoic acid, (E) isobutyric acid, (F) 2-methylbutyric acid in male and female C57BL/6 mice exposed to 35 μg diesel exhaust particles (DEP) suspended in saline or sterile saline control, administered 2×/week for 50 days, and treated with placebo (PLC) or probiotic (PRO). Data are presented as mean ± SEM with individual animals overlaid. Statistical significance was determined by three-way ANOVA followed by Holm–Šídák’s multiple comparisons test. *p < 0.05, **p < 0.01, ***p < 0.001, ****p < 0.0001.

Three-way ANOVA revealed effects of sex, probiotic treatment, and DEP exposure on circulating SCFAs and branched chain fatty acids (BCFAs) (Fig. 7). Acetic acid (Fig. 7A) showed significant main effects of probiotic treatment (F(1,53) = 83.70, *p* < 0.0001, ηp^2^ = 0.61) and exposure (F(1,53) = 8.59, *p* = 0.0050, ηp^2^ = 0.14), as well as significant sex × probiotic (F(1,53) = 5.71, *p* = 0.0205, ηp^2^ = 0.10), sex × exposure (F(1,53) = 9.93, *p* = 0.0027, ηp^2^ = 0.16), probiotic × exposure (F(1,53) = 5.25, *p* = 0.0259, ηp^2^ = 0.09), and sex × probiotic × exposure (F(1,53) = 10.56, *p* = 0.0020, ηp^2^ = 0.17) interactions.

3-hydroxybutyric acid (Fig. 7B) was significantly influenced by sex (F(1,55) = 46.40, p < 0.0001, ηp^2^ = 0.46) and probiotic treatment (F(1,55) = 48.38, p < 0.0001, ηp^2^ = 0.47). Post hoc comparisons indicated that males generally exhibited higher levels than females, including higher levels in male SAL + PLC versus female SAL + PLC (p = 0.0235) and in male DEP + PLC versus female DEP + PLC (p = 0.0002). Among females, probiotic treatment increased 3-hydroxybutyric acid, with female SAL + PRO (p = 0.0098) and female DEP + PRO (p = 0.0423) higher than female SAL + PLC.

Isovaleric acid (Fig. 7C) showed significant main effects of sex (F(1,55) = 6.99, *p* = 0.0106, ηp^2^ = 0.11), probiotic treatment (F(1,55) = 5.75, *p* = 0.0199, ηp^2^ = 0.09), and exposure (F(1,55) = 4.38, *p* = 0.0410, ηp^2^ = 0.07), along with a significant sex × exposure interaction (F(1,55) = 6.08, *p* = 0.0168, ηp^2^ = 0.10). Post hoc differences were more limited, although female DEP + PLC mice differed from female SAL + PRO mice (*p* = 0.0075), suggesting more modest and group-specific effects.

Hexanoic acid (Fig. 7D) was strongly affected by sex (F(1,55) = 32.15, *p* < 0.0001, ηp^2^ = 0.37) and showed significant sex × probiotic (F(1,55) = 16.37, *p* = 0.0002, ηp^2^ = 0.23) and sex × probiotic × exposure (F(1,55) = 11.01, *p* = 0.0016, ηp^2^ = 0.17) interactions. Post hoc analysis indicated that males generally exhibited higher hexanoic acid levels than females, including higher levels in male DEP + PLC relative to female SAL + PLC, female DEP + PLC, and female SAL + PRO groups (all *p* < 0.0001). Within females, DEP + PRO mice showed higher hexanoic acid levels than DEP + PLC mice (*p* = 0.0185).

Isobutyric acid (Fig. 7E) was significantly influenced by sex (F(1,54) = 11.62, *p* = 0.0012, ηp^2^ = 0.18), with males exhibiting higher circulating levels than females overall. A significant sex × exposure interaction (F(1,54) = 4.20, *p* = 0.0453, ηp^2^ = 0.07) indicated that DEP exposure decreased isobutyric acid in males while slightly increasing it in females.

2-Methylbutyric acid (Fig. 7F) showed a strong main effect of sex (F(1,53) = 23.36, *p* < 0.0001, ηp^2^ = 0.31), with males higher than females across all treatment groups. A significant sex × exposure interaction (F(1,53) = 4.43, *p* = 0.0400, ηp^2^ = 0.08) reflected a DEP-associated decrease in males that was not observed in females.

Butyric acid (Figure S1A) and 4-Methylvaleric acid (Figure S1B) levels were also quantified in the plasma samples; however, no statistical differences were noted across exposures, treatment, or sex groups. Total SCFA and BCFA (Figure S2) analysis shows a significant main effects of sex (F(1,51) = 40.87, p < 0.0001, ηp^2^ = 0.45; higher in males) and probiotic treatment (F(1,51) = 42.64, p < 0.0001, ηp^2^ = 0.46; higher with probiotic in both sexes); the main effect of exposure and all interaction terms were not significant (all p > 0.11). However, because 3-hydroxybutyric acid circulates at substantially higher concentrations than the other five analytes, this composite total is numerically dominated by 3-hydroxybutyric acid (Fig. 7B) and thus closely mirrors its quantification pattern.

### Circulating LPS and blood glucose levels were significantly influenced by sex, DEP exposure, and probiotic treatment.

**Figure 8:**
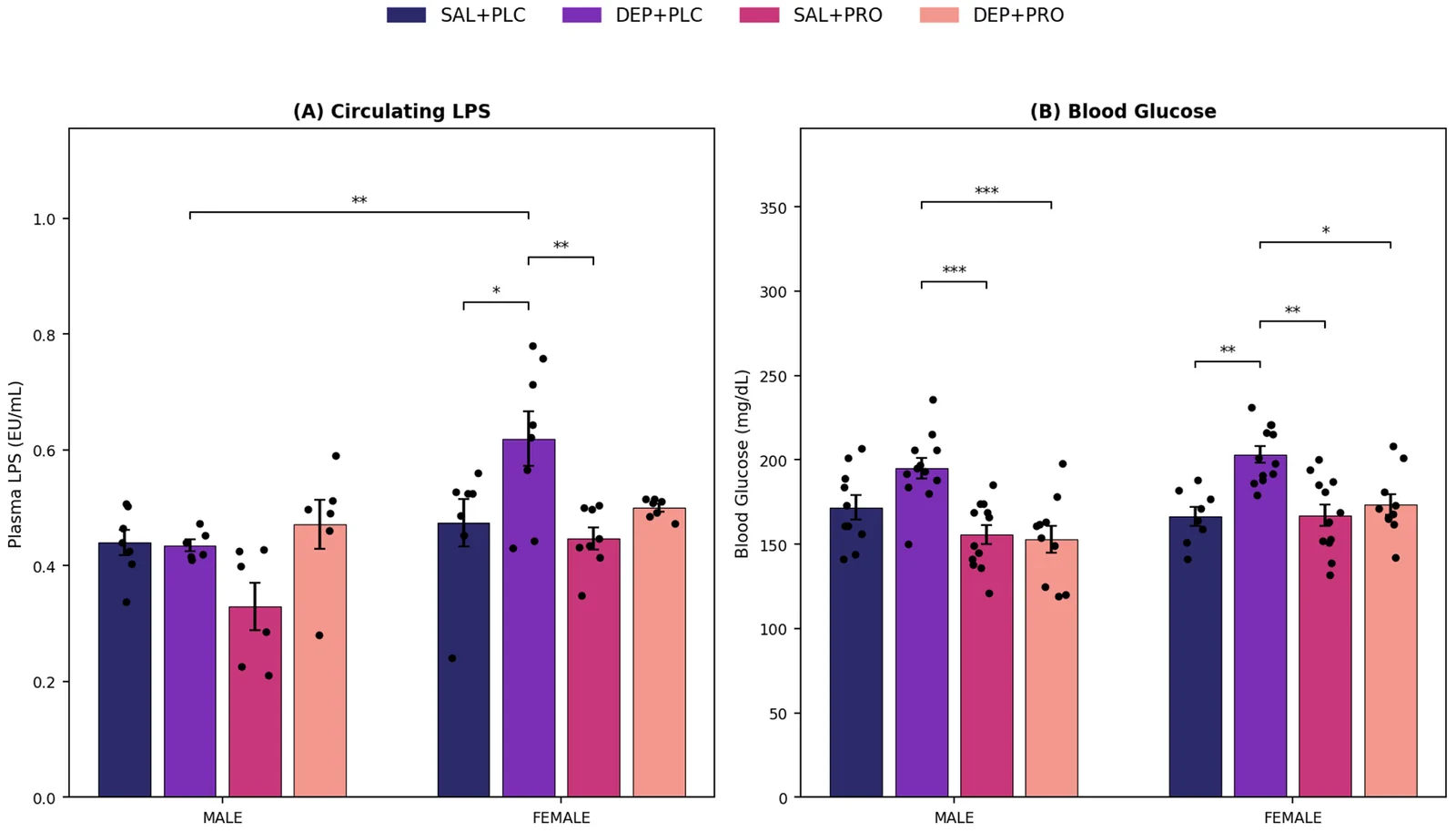
Plasma LPS and blood glucose levels across exposure, treatment, and sex. Circulating plasma (A) lipopolysaccharide (LPS) concentration (EU/mL) in male and female C57BL/6 mice exposed to diesel exhaust particles (DEP) or saline (SAL) and treated with placebo (PLC) or probiotic (PRO). (B) Blood glucose concentration (mg/dL) in the same groups. Mice were exposed to 35 μg DEP suspended in saline or sterile saline control, administered 2×/week for 50 days. Data are presented as mean ± SEM with individual animals overlaid. Statistical significance was determined by three-way ANOVA followed by Holm–Šídák’s multiple comparisons test. *p < 0.05, **p < 0.01, ***p < 0.001, ****p < 0.0001.

Figure 8A shows the circulating plasma concentration (EU/mL) of LPS. Three-way ANOVA revealed significant main effects of sex (F(1,47) = 15.31, p = 0.0003, ηp^2^ = 0.25), exposure (F(1,47) = 13.10, p = 0.0007, ηp^2^ = 0.22), and probiotic treatment (F(1,47) = 5.60, p = 0.0221, ηp^2^ = 0.11), as well as a significant sex × probiotic × exposure interaction (F(1,47) = 6.68, p = 0.0129, ηp^2^ = 0.12), indicating sex-dependent treatment effects. Post hoc analyses revealed distinct female-specific responses. In females, DEP exposure significantly increased circulating LPS compared with saline controls (SAL + PLC vs. DEP + PLC, *p* = 0.0431). No significant differences were observed between DEP + PLC and DEP + PRO females (*p* = 0.171). In males, no significant within-sex treatment differences were detected. However, several between-sex comparisons were significant, with females exhibiting higher LPS levels than males under DEP exposure conditions, including DEP + PLC (*p* = 0.0059). These results suggest that DEP’s effect on LPS is sex dependent.

Blood glucose levels are also shown in Fig. 8B. Three-way ANOVA revealed significant main effects of probiotic treatment (F(1,78) = 23.55, *p* < 0.0001, ηp^2^ = 0.23) and exposure (F(1,78) = 12.61, *p* = 0.0007, ηp^2^ = 0.14), as well as a significant probiotic × exposure interaction (F(1,78) = 9.75, *p* = 0.0025, ηp^2^ = 0.11), indicating that the effect of DEP on glucose levels differed depending on probiotic treatment. No significant main effect of sex (F(1,78) = 3.86, p = 0.0530) or sex-dependent interactions were observed.

Interestingly, post hoc analysis demonstrated that DEP increased blood glucose in placebo-treated females, with female DEP + PLC mice exhibiting higher glucose levels than female SAL + PLC (*p* = 0.0050). This increase was attenuated by probiotic treatment, as female DEP + PRO mice showed lower glucose levels than DEP + PLC (*p* = 0.0269). In males, DEP exposure alone did not significantly alter glucose levels in placebo groups (*p* = 0.1615); however, male DEP + PLC mice exhibited higher glucose compared to both male SAL + PRO (*p* = 0.0003) and male DEP + PRO (*p* = 0.0002) groups, suggesting a protective effect of probiotic treatment under DEP exposure conditions. Several between-sex differences were also observed, with female DEP + PLC mice exhibiting higher glucose levels than multiple male groups, including male SAL + PLC (*p* = 0.0138), male SAL + PRO (*p* < 0.0001), and male DEP + PRO (*p* < 0.0001).

### Metabolic and inflammatory biomarkers reveal sex-specific host responses to exposure.

**Figure 9:**
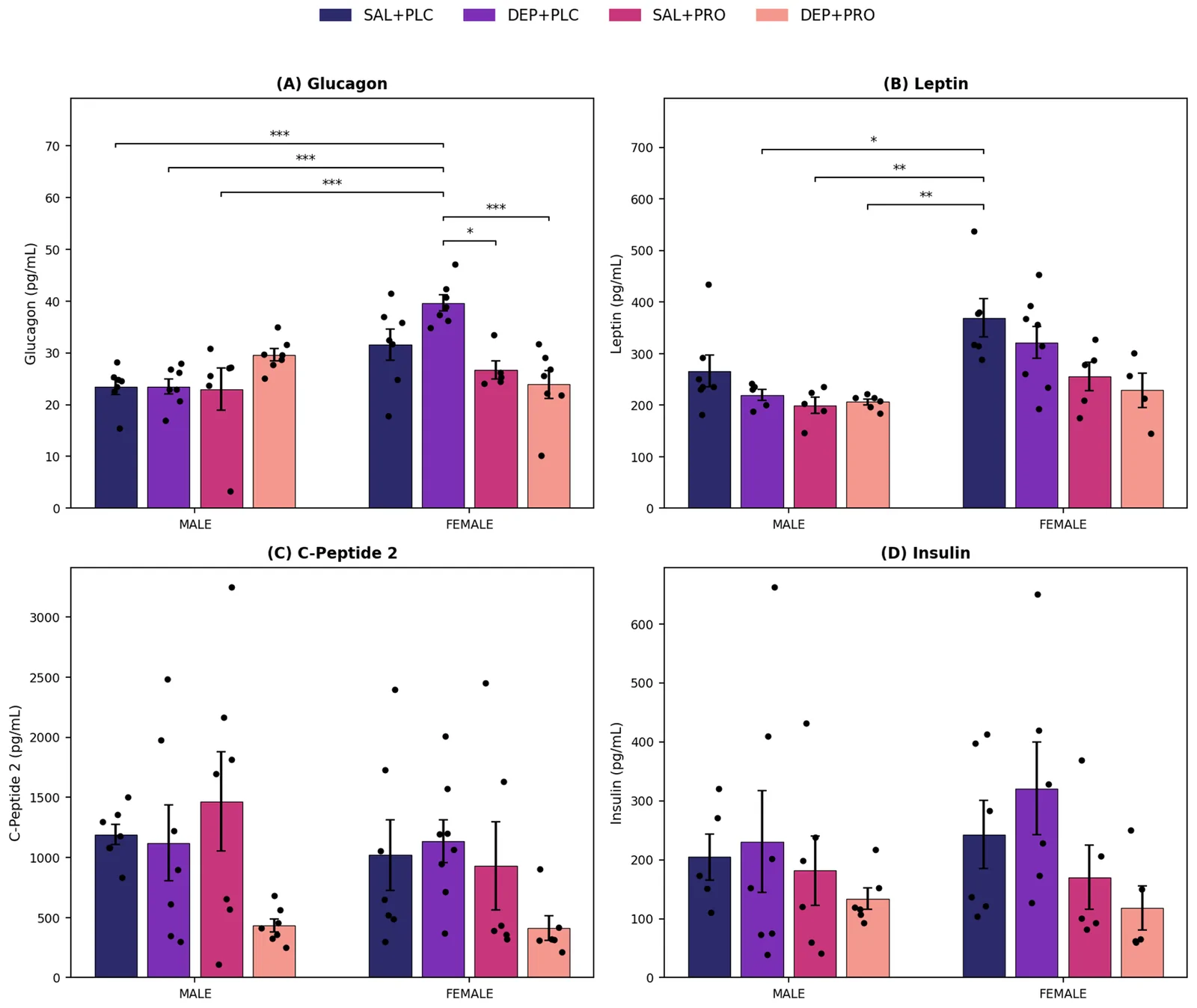
Plasma metabolic hormone concentrations across exposure, treatment, and sex. Circulating plasma concentrations of metabolic hormones in male and female C57BL/6 mice following diesel exhaust particle (DEP) exposure and probiotic treatment. Panels show (A) glucagon, (B) leptin, (C) c-peptide 2, and (D) insulin in mice exposed to 35 μg DEP suspended in saline or sterile saline control, administered 2×/week for 50 days, and treated with placebo (PLC) or probiotic (PRO). Data are presented as mean ± SEM with individual animals overlaid. Statistical significance was determined by three-way ANOVA followed by Holm–Šídák’s multiple comparisons test. *p < 0.05, **p < 0.01, ***p < 0.001, ****p < 0.0001.

Three-way ANOVA revealed hormone-specific effects of sex, probiotic treatment, and DEP exposure on circulating metabolic hormones (Fig. 9). Glucagon was significantly influenced by sex (F(1,45) = 11.57, *p* = 0.0014, ηp^2^ = 0.20), with females presenting higher concentrations. Three-way ANOVA revealed significant main effects with probiotic treatment (F(1,45) = 5.25, *p* = 0.0267, ηp^2^ = 0.10), significant sex × probiotic (F(1,45) = 16.10, *p* = 0.0002, ηp^2^ = 0.26) and sex × probiotic × exposure (F(1,45) = 7.11, *p* = 0.0106, ηp^2^ = 0.14) interactions (Fig. 9A). The interaction revealed glucagon increased with probiotic treatment in males but decreased in females. Post hoc analysis showed that female DEP + PLC mice exhibited higher glucagon levels than several male groups and higher levels than both female SAL + PRO (*p* = 0.0126) and female DEP + PRO (*p* = 0.0003), suggesting a probiotic-associated attenuation in DEP-exposed females. For leptin (Fig. 9B), post hoc differences were limited, although female SAL + PLC mice exhibited higher levels than several male groups, supporting an overall sex-dependent effect. Interestingly, C-peptide 2 (Fig. 9C), decreased with exposure in probiotic treated groups, with a significant main effect of exposure (F(1,47) = 4.22, *p* = 0.0455, ηp^2^ = 0.08) and a significant probiotic × exposure interaction (F(1,47) = 4.75, *p* = 0.0343, ηp^2^ = 0.09). Insulin (Fig. 9D), showed a significant main effect of probiotic treatment only (F(1,38) = 5.09, *p* = 0.0299, ηp^2^ = 0.12), whereas leptin was significantly affected by sex (F(1, 38) = 12.37, *p* = 0.0011, ηp^2^ = 0.25), with females showing higher baseline concentrations, and probiotic treatment (F(1,38) = 12.60, *p* = 0.0010, ηp^2^ = 0.25), with probiotic significantly decreasing concentrations.

**Figure 10:**
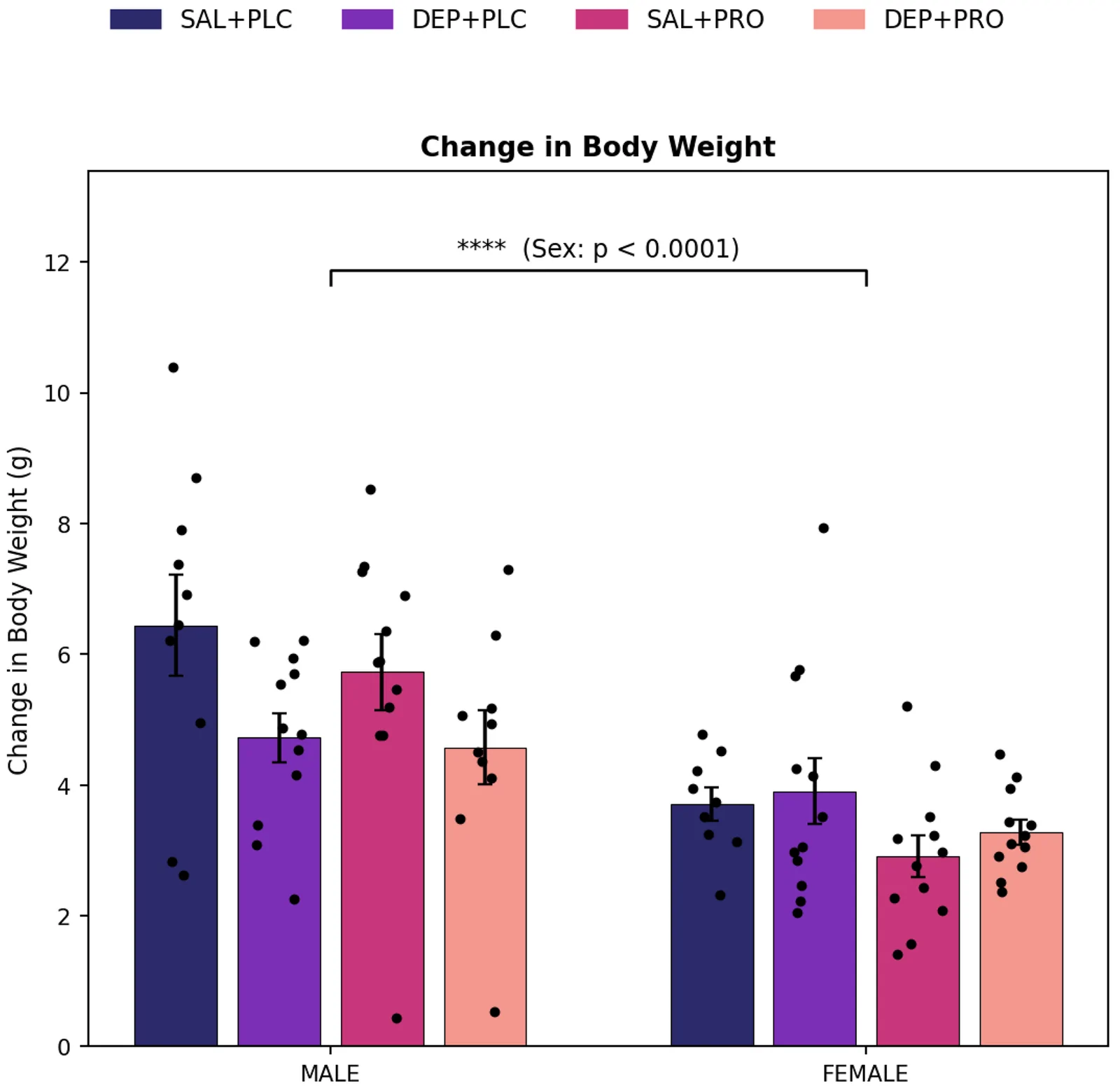
Change in group body weight across exposure, treatment, and sex. Change in body weight in male and female C57BL/6 mice following diesel exhaust particle (DEP) exposure and probiotic treatment. Mice were exposed to 35 μg DEP suspended in saline or sterile saline control, administered 2×/week for 50 days, and treated with placebo (PLC) or probiotic (PRO). Graph shows change in body weight from exposure day 0 to day 50. Data are presented as mean ± SEM with individual animals overlaid. Statistical significance was determined by three-way ANOVA followed by Holm–Šídák’s multiple comparisons test. ****p < 0.0001.

Additionally, total change in body weight (Fig. 10) was significantly influenced by sex (F(1,81) = 32.24, *p* < 0.0001, ηp^2^ = 0.28) and showed a significant sex × exposure interaction (F(1,81) = 6.45, *p* = 0.0130, ηp^2^ = 0.07), with no significant effects of probiotic treatment (F(1,81) = 2.87, p = 0.0943). The sex x exposure interaction indicates that DEP exposure was associated with reduced weight gain in males, but not females, though the within-sex pairwise comparison (SAL + PLC vs. DEP + PLC) did not reach significance (p = 0.2116). This likely reflects the higher variance in the male SAL + PLC group.

## Discussion

Understanding sex-related differences in gut-mediated outcomes following environmental exposure is essential for developing targeted interventions. Overall, the present study demonstrates that inhaled DEP alters gut microbial composition, circulating microbial-associated metabolites, and host metabolic biomarkers in a sex-dependent manner, with probiotic treatment modifying several of these responses.

To quantify observations in diversity of the gut microbiome, we employed several metrics. Alpha diversity metrics, such as Chao1, ACE, Shannon, and Simpson Evenness, capture distinct features of community structure. Chao1 and ACE are richness estimators, meaning they quantify the total number of taxa present. Simpson Evenness measures the relative taxon abundances, independent of how many taxa are present. Shannon takes it a step further and integrates both richness and evenness into a singular measurement. Female mice exhibited significant reductions in Shannon diversity and a trend toward reduced evenness in DEP-exposed placebo animals compared to saline controls. This indicated that DEP exposure was associated with a shift towards a less complex, less evenly distributed community structure. This pattern is associated with increased susceptibility to further perturbation (31). Male mice demonstrated significant overall differences in richness and evenness, driven primarily by markedly expanded species richness in DEP + PRO animals compared to DEP + PLC. This suggests that probiotic supplementation produces particularly pronounced microbiome expansion in males under DEP exposure conditions. β-diversity analysis revealed significant treatment-associated differences in community composition in females, with a trending effect in males.

Beyond observations of diversity indices, DEP exposure did alter microbiota at the taxonomic level with known functional relevance. Specifically, DEP exposure induced a restructuring of the gut microbiome characterized by increased abundance of *Desulfovibrio*, a gram-negative genus, associated with hydrogen sulfide production, LPS generation, and impaired gut barrier integrity (18, 32). In female mice, the probiotic treatment reduced *Muribaculaceae* abundance in the saline control groups. Although *Muribaculaceae* is generally considered to be a commensal or beneficial microbe for its role in carbohydrate fermentation and the production of SCFAs, the observed reduction may reflect competitive or functional displacement (33). This means that another taxon may fill in this role, restructuring the community, but preserving the metabolic output. Additionally, beneficial taxa such as *Clostridium sensu stricto 1*, which is associated with metabolic regulation and SCFA production, were reduced in females following both DEP exposure and probiotic treatment (34). *Turicibacter*, associated with metabolic regulation and SCFA production, was reduced in SAL + PRO females but notably elevated in DEP + PRO females, suggesting that the probiotic effect on this genus is context-dependent and modified by DEP exposure (35). *Turicibacter* is of interest because it has been shown to promote metabolic health by reducing weight gain and lowering fasting blood glucose (36). Some interesting genera, including *Desulfovibrio* and *Enterorhabdus*, increased with probiotic treatment in females but decreased in males, further supporting sex-dependent differences in microbiome response. Together, these findings indicate that microbiome composition and its response to environmental and probiotic interventions are inherently sex-dependent, likely reflecting underlying differences in hormonal, metabolic, and immune regulation.

Consistent with these compositional shifts, circulating fatty-acid metabolites were differentially affected across treatment groups. DEP significantly increased LPS, consistent with increased endotoxemia and possible disruption of gut barrier integrity (37). This effect was more pronounced in female mice. Circulating fatty-acid profiles also demonstrated sex-dependent variation. Both 3-hydroxybutyric acid and hexanoic acid differed significantly by sex, reflecting their position at the intersection of fat metabolism, hormonal regulation and microbial activity (38). Probiotic treatment was also a significant driver of variation in 3-hydroxybutyric acid levels, suggesting an effect of microbiome modulation on host energy metabolism. In contrast, hexanoic acid was not significantly altered by probiotics alone, but instead was influenced through interaction effects with sex and exposure, indicating greater sensitivity to combined host and environmental influences. Acetic acid concentrations were not independently driven by sex; however, sex modified the effects of DEP exposure and probiotic treatment. This suggests that acetate production may be highly sensitive to both environmental and microbial influences. We observed similar effects on the branched-chain fatty acids (BCFAs) isobutyric acid and 2-methylbutyric acid, which are produced primarily through bacterial fermentation of branched-chain amino acids rather than through carbohydrate fermentation (39). Both BCFAs were significantly higher in males than females and exhibited significant sex x exposure interactions, with DEP exposure decreasing their circulating concentrations in males, and having little effect in females. This pattern parallels the male-specific response observed for hexanoic acid and suggests that DEP disrupts microbial and host fatty-acid metabolism differentially between sexes. Together, these findings suggest that microbiome-driven alterations in circulating metabolites may contribute to downstream systemic inflammatory and metabolic changes, prompting further evaluation of circulating biomarkers.

To explore the impact that these circulating microbial-associated and host-derived metabolites may have on health outcomes in our mice, we assessed biomarkers of metabolic function. Analysis of these biomarkers revealed clear sex-, exposure-, and treatment-dependent effects. Notably, in females, DEP exposure significantly elevated glucagon in placebo-treated animals compared to saline controls, suggesting DEP-induced dysregulation of glucoregulatory signaling (40). However, this elevation is attenuated by probiotic supplementation. Interestingly, DEP exposure alone did not alter glucagon levels in males; however, we observed elevated glucagon in the DEP + PRO treatment group. This finding supports a role for microbiome modulation in restoring metabolic balance in response to environmental exposure and underscores the importance of sex as a biological variable to consider in intervention strategies. Blood glucose measurements also supported this pattern, with DEP-induced metabolic disruption more pronounced in females and partially mitigated by probiotic intervention. In males, DEP exposure alone did not significantly alter glucose levels. However, probiotic treatment was associated with lower glucose under both exposure conditions. Insulin levels were influenced by both probiotic treatment and sex, with females showing higher insulin levels independent of DEP exposure. These biomarker-specific differences suggest that DEP exposure and probiotic treatment do not uniformly affect metabolic pathways, but rather selectively influence distinct physiological systems.

Together, these findings support the idea that inhaled DEP exposure can influence host physiology through gut-associated pathways, with sex acting as an important modifier of these effects. Probiotic treatment modified several DEP-associated physiological outcomes, with the most pronounced attenuation observed in females for inflammatory and glucoregulatory markers, while microbiome compositional effects were more pronounced in males. These findings suggest that microbiome-targeted strategies could reduce metabolic risk in populations with high TRAP-PM exposure, particularly among females, who appear to be disproportionately affected.

There are, however, limitations of this study that should be considered. Although 16S sequencing allowed us to characterize changes in microbial composition, these changes were only observed at a single time point. Future investigation may include samples collected at multiple points throughout the exposure period. Additionally, gut barrier integrity was not assessed, limiting conclusions regarding intestinal permeability and its contribution to systemic effects. Finally, because the study was limited to a single exposure dose and time point, the findings may not fully reflect the dose-dependent or longitudinal effects of DEP exposure and probiotic treatment. The DEP dose exceeds typical ambient exposure levels, with alveolar deposition ~ 40-fold higher than that from a 24-hour human exposure at 100 μg/m^3^ (41). However, this dose was selected based on prior studies demonstrating reproducible pulmonary and cardiovascular effects (14, 37, 41).

Taken together, these findings reframe air pollution exposure as a gut-mediated metabolic stressor whose impact is fundamentally shaped by biological sex, a variable that has been historically underrepresented in environmental health research. Future work should focus on investigating the mechanistic basis of sex-specific responses, incorporating environmentally relevant exposure models, and evaluating gut barrier function and microbial metabolism to better define the causal relationships between inhaled particulate matter, the microbiome, and host health outcomes.

## Conclusions

Our findings demonstrate that inhaled DEP exposure alters gut microbial composition and host metabolic signaling in a sex-dependent manner. DEP exposure was associated with shifts in microbial diversity and taxonomic composition, altered circulating LPS and glucose, and changes in SCFAs and metabolic hormones, several of which were modified by probiotic treatment. These findings are particularly important for understanding how inhaled particulate exposure may influence host physiology through gut-associated pathways and how sex may shape these responses. The use of probiotics in this study helped define the extent to which microbiome-targeted intervention can modulate selected effects of environmental PM exposure, while also highlighting that these responses are not uniform across outcomes. Thus, our findings provide a foundation for future studies investigating the mechanisms linking inhaled pollutants to gut-microbiome-host signaling and offer insight into the therapeutic potential of microbiome-targeted strategies for mitigating PM exposure-driven metabolic dysfunction.

## Supplementary Material

This is a list of supplementary files associated with this preprint. Click to download.

- YoungbloodBoSDSupplementaryFile.docx

## Figures and Tables

**Table 1:** Exposure and treatment groups

Female C57BL/6	Exposure	Treatment	Group
	
4–6 weeks old	Saline	Placebo	SAL + PLC (n = 12)
Female C57BL/6 4–6 weeks old	Saline	Probiotic	SAL + PRO (n = 12)

Female C57BL/6 4–6 weeks old	DEP	Placebo	DEP + PLC (n = 12)

Female C57BL/6 4–6 weeks old	DEP	Probiotic	DEP + PRO (n = 12)

Male C57BL/6 4–6 weeks old	Saline	Placebo	SAL + PLC (n = 12)

Male C57BL/6 4–6 weeks old	Saline	Probiotic	SAL + PRO (n = 12)

Male C57BL/6 4–6 weeks old	DEP	Placebo	DEP + PLC (n = 12)

Male C57BL/6 4–6 weeks old	DEP	Probiotic	DEP + PRO (n = 12)

Male and female C57BL/6 mice (4–6 weeks of age at study initiation) were randomized to receive saline or diesel exhaust particle (DEP) exposure in combination with placebo (PLC) or probiotic (PRO) treatment, resulting in four treatment groups per sex (n = 12/group).

## Data Availability

The datasets generated and/or analyzed during the current study are publicly available in Mendeley Data repository, DOI: 10.17632/jbxzng6755.1.

## Highlights

- Inhaled diesel exhaust particles (DEP) alter gut microbial composition and circulating metabolites in a sex-dependent manner, with greater diversity loss in females and stronger probiotic-driven community expansion in males.
- DEP exposure increased circulating lipopolysaccharide (LPS) and disrupted blood glucose regulation, with more pronounced effects in females that were partially attenuated by probiotic treatment.
- Probiotic supplementation modifies glucagon levels in a sex-specific manner, eliciting opposing response patterns in males and females.
- Circulating concentrations of short- and branched-chain fatty acids, such as 3-hydroxybutyric, hexanoic, isobutyric, and 2-methylbutyric acids, are consistently higher in males, indicating a broader male-specific metabolic signature.
- These findings underscore the importance of incorporating sex as a biological variable in environmental health research and suggest that microbiome-targeted interventions may provide sex-specific protection against air pollution-induced metabolic dysfunction.
